# Interlayer Tailoring of Ti–6Al–4V and 17-4PH Stainless Steel Joint by Tungsten Inert Gas Welding

**DOI:** 10.3390/ma16124370

**Published:** 2023-06-14

**Authors:** Raj Narayan Hajra, Chan Woong Park, Kyunsuk Choi, Jeoung Han Kim

**Affiliations:** 1 Department of Materials Science & Engineering, Hanbat National University, Daejeon 34158, Republic of Korea; rajjhajra@gmail.com (R.N.H.);; 2 Department of Industry-University Convergence, Hanbat National University, Daejeon 34158, Republic of Korea

**Keywords:** Ti–6Al–4V, 17-4PH, tungsten inert gas welding, interlayers, elemental separation

## Abstract

The development of robust and efficient methods for constructing and joining complex metal specimens with high bonding quality and durability is of paramount importance for various industries, e.g., aerospace, deep space, and automobiles. This study investigated the fabrication and characterization of two types of multilayered specimens prepared by tungsten inert gas (TIG) welding: Ti–6Al–4V/V/Cu/Monel400/17-4PH (Specimen 1) and Ti–6Al–4V/Nb/Ni–Ti/Ni–Cr/17-4PH (Specimen 2). The specimens were fabricated by depositing individual layers of each material onto a Ti–6Al–4V base plate, and subsequently welding them to the 17-4PH steel. The specimens exhibited an effective internal bonding without any cracks, accompanied by a high tensile strength, with Specimen 1 exhibiting a significantly higher tensile strength than Specimen 2. However, the substantial interlayer penetration of Fe and Ni in the Cu and Monel layers of Specimen 1 and the diffusion of Ti along the Nb and Ni–Ti layers in Specimen 2 resulted in a nonuniform elemental distribution, raising concerns about the lamination quality. This study successfully achieved elemental separation of Fe/Ti and V/Fe, which is vital for preventing the formation of detrimental intermetallic compounds, particularly in the fabrication of complex multilayered specimens, representing the prime novelty of this work. Our study highlights the potential of TIG welding for the fabrication of complex specimens with high bonding quality and durability.

## 1. Introduction

Titanium alloys and stainless steels are two of the most widely used materials in many critical applications owing to their superior mechanical properties, excellent corrosion resistance, and biocompatibility. Welding these materials is of great interest in many industries, including the aerospace, marine, and biomedical fields [1,2,3,4,5,6,7]. However, welding titanium and stainless steel alloys is challenging due to their inherently different metallurgical properties, which can lead to the formation of brittle intermetallic compounds (IMCs) and thermal distortion during welding [8]. The most damaging phase among various Ti-based IMCs is TiFe_2_. Wang et al. [9] reported the spontaneous cracking of a Ti alloy/304 stainless steel joint due to the formation of TiFe_2_ and TiFe brittle phases. Chen et al. [10] observed a significant decrease in the number of brittle TiFe_2_ phases in the weld seam by offsetting the laser beam toward the stainless steel during laser welding, resulting in a durable joint with a tensile strength of 150 MPa. Xia et al. [11] found that the lattice mismatch between TiFe and TiFe_2_ induced cracks through the brittle TiFe_2_ layer in a brazed Ti alloy/316L stainless steel joint. These studies suggest that suppressing the formation of TiFe and TiFe_2_ brittle phases is crucial for a successful joining of Ti alloys and steels, which are dissimilar materials. To achieve this, solid-state welding techniques, such as diffusion bonding, friction welding, and explosive welding, are preferred [12,13]. For example, Kahraman et al. [14] produced a sound Ti/stainless steel joint with a superior tensile strength of 726 MPa using explosive welding. Dong et al. [15] produced a high-quality TC4 Ti alloy/40Cr steel friction-welded joint with a tensile strength of 766 MPa and a bending angle of 32.5°. Brazing is another suitable option for the dissimilar material joining of Ti alloys and stainless steels owing to the good controllability of the interfacial reaction, low joining temperature, and low residual stress. Xia et al. [11,16] designed innovative amorphous filler metals to braze Ti alloys and 316 L stainless steel, resulting in an optimized shear strength.

There is growing demand for the fusion welding of Ti alloys and stainless steels owing to its flexibility in shaping the weldment and its high production efficiency. However, direct fusion welding is not viable for joining Ti alloys and stainless steel since the resulting joint tends to fracture spontaneously, even when high-energy beam welding techniques, such as laser or electron beam welding, are utilized [9,10]. Current literature on the fusion welding of titanium and stainless steel alloys has shown that the use of interlayers significantly influences the quality and mechanical properties of welded joints. The effects of different interlayers on the microstructures and mechanical properties of fusion-welded Ti alloys and stainless steel have been evaluated. For instance, Zhang et al. [17,18,19] investigated the effects of various interlayers on the mechanical properties of Ti and steel joints. Pugacheva et al. [20] conducted laser welding of pure titanium and stainless steel using a 1 mm thick pure copper sheet as an interlayer. The formation of (Fe,Cr)_2_Ti was effectively inhibited, and the (Fe,Cr)_2_Ti and Cu_3_Ti nanoparticles were homogeneously dispersed in the supersaturated Cu-based weld. However, the conventional joints and specific welding parameters of the aforementioned solid-state welding methods may limit their widespread application. To overcome these challenges, a reliable and efficient welding method is required for joining these materials while preserving their properties.

The motivation for designing multilayered specimens stems from the growing interest in high-entropy alloys (HEAs), which have demonstrated exceptional mechanical properties and unique microstructures. HEAs, which are characterized by their composition of multiple elements in near-equiatomic proportions, have attracted attention for their potential in various applications. In line with the concept of creating alloy systems with enhanced properties, the present study explores the fabrication and characterization of multilayered specimens using TIG welding. In recent years, TIG welding has emerged as a promising welding technique for joining titanium and stainless steel alloys owing to its high precision, low heat input, and minimal distortion. The economic benefits of high-quality arc welding for joining Ti alloys and stainless steel, specifically TIG and metal inert gas (MIG) welding, are considerable due to the wide applicability, low cost, and potential for automated production of these types of welding. However, the TIG welding of Ti and stainless steel joints tends to produce cracks, particularly under high welding heat inputs [21,22]. Residual welding stress is a critical factor that can lead to crack initiation, ultimately limiting the mechanical properties of the joint [23,24]. Moreover, three major issues remain unresolved: (i) distribution of residual stress in Ti/steel joints; (ii) effect of the filler metal on residual stress; and (iii) optimization of welding parameters to minimize residual stress.

This study aimed to address these research gaps by investigating the effects of various interlayers on the formation of IMCs, microstructure, and mechanical properties of TIG-welded Ti–6Al–4V and 17-4PH joints. The fabrication process of Ti–6Al–4V and 17-4PH stainless steels was innovatively designed using TIG welding with the following sequences: (i) Ti–6Al–4V/V/Cu/Monel 400/17-4PH and (ii) Ti–6Al–4V/Nb/Ni–Ti/Ni–Cr/17-4PH.

## 2. Materials and Methods

**Figure 1:**
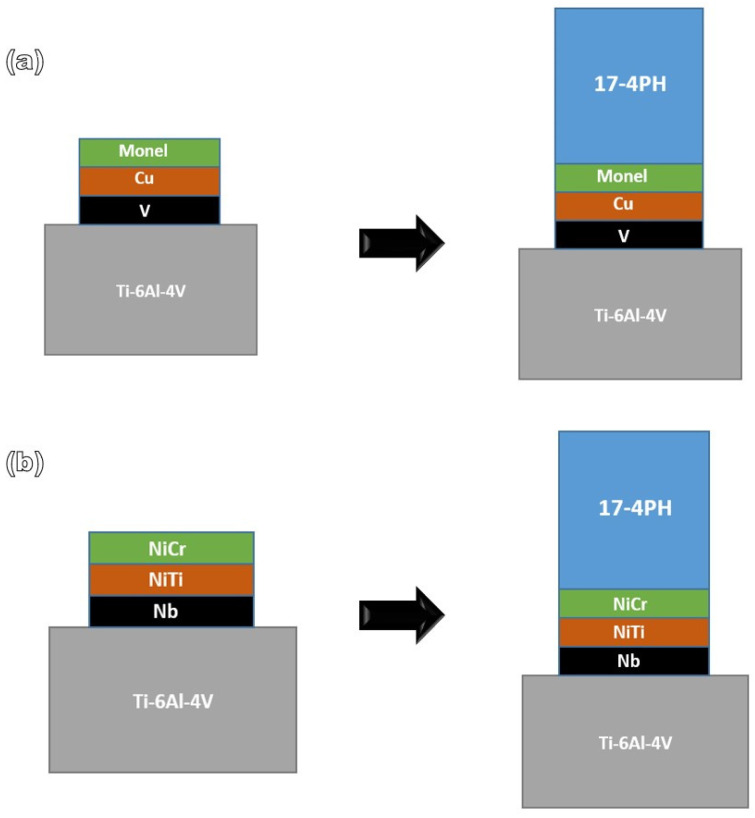
Schematics of the fabrication strategy: (**a**) Deposition of two layers of V, Cu, and Monel by TIG torch; grinding of the upper layer; and finally, welding of the 17-4PH layers; (**b**) deposition of two layers of each interlayer of Nb, Ni–Ti, and Ni–Cr prior to welding.

Figure 1 shows the schematics of the fabrication sequence used for the complex construction of Specimen 1, which is made of Ti–6Al–4V/V/Cu/Monel 400/17-4PH. A Ti–6Al–4V base plate with dimensions of 110 mm × 110 mm × 35 mm was used. The base plate surface was ground, cleaned with alcohol, and then cleaned ultrasonically. First, two layers of V with dimensions of 20 mm × 12 mm were deposited using a hand TIG torch. This was followed by the deposition of two layers of Cu and two layers of Monel 400. Finally, TIG welding was performed on the deposited Ti–6Al–4V with 17-4PH, as illustrated in Figure 1.

All the materials used in the TIG welding experiments were provided by Thermo Fisher Scientific. The experiments were conducted under argon gas flow. The fabrication process was repeated several times to ensure good repeatability. The second specimen was fabricated using two layers of Nb, two layers of Ni–Ti, and one layer of Ni–Cr. The TIG-welded specimens were cut to observe the cross-sectional region and ground using a SiC paper. The specimens were polished on a felt disk with 3, 1, and 0.25 μm diamond suspensions and colloidal silica until they exhibited a mirror-like surface. Finally, the specimens were ultrasonically cleaned with ethyl alcohol. The detailed microstructures of the interfaces of the interlayers were analyzed by field-emission scanning electron microscopy (FE-SEM; HITACHI SU5000, Japan) with an energy dispersive spectrometer (EDS). The mechanical properties, such as the tensile strength and Vickers hardness, were evaluated to test the manufacturing durability. The tensile specimens were prepared in order that the copper layer is located in the middle, and tensile tests were conducted at a rate of 10^−3^ s^−1^ at room temperature using an INSTRON–5982 instrument. The Vickers hardness was examined with a dwell time of 10 s and a load of 100 kgf (Struers; Duramin–100).

## 3. Results

### 3.1. Microstructural Characterization

**Figure 2:**
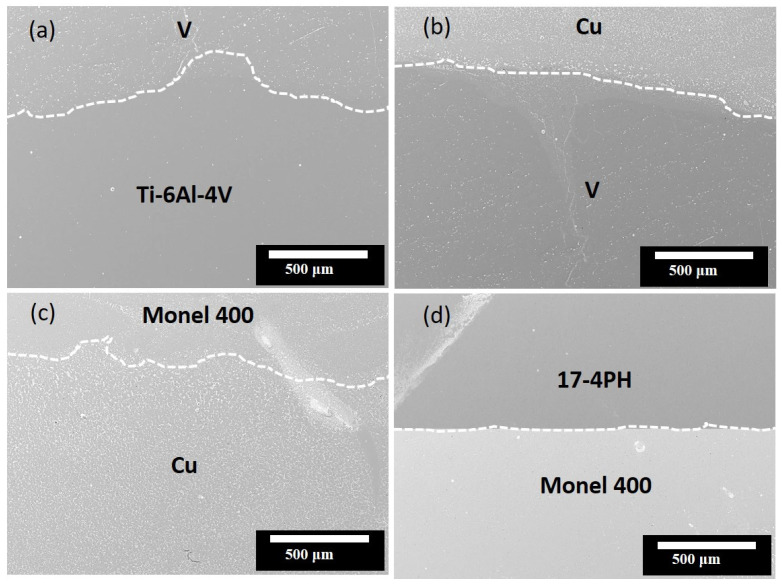
SEM microstructures of the morphology and cross-sectional images of the fabrication of (**a**) Ti–6Al–4V/V, (**b**) V/Cu, (**c**) Cu/Monel 400, and (**d**) Monel 400/17-4PH.

Figure 2 shows the morphology and cross-sectional image of Specimen 1, along with the individual layers of V, Cu, and Monel, which are seen to be effectively bonded internally.

**Figure 3:**
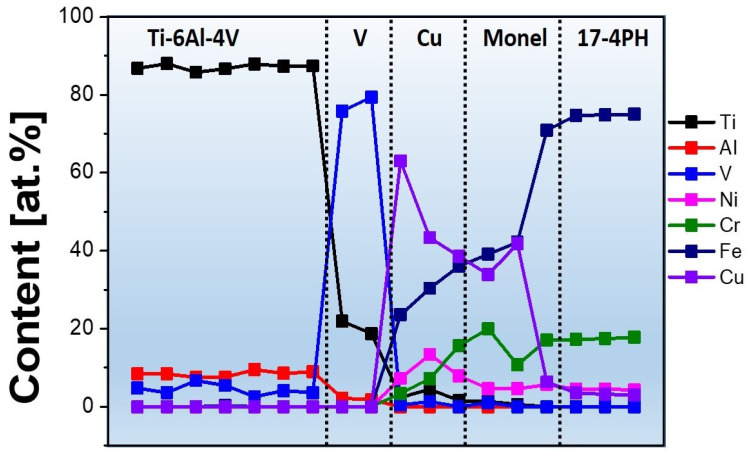
Elemental distribution throughout the interlayers of Specimen 1, obtained by EDS.

The bonding between the interlayers and dissimilar terminal alloys was crack-free, which is the primary achievement of this study. Notably, the thickness of the individual layers varied from 800 to 1200 μm. Figure 3 shows the elemental distribution along the fabrication route of the transition metal as analyzed by EDS. Eighteen points were uniformly measured along the route, with a dwell time of 10 min for each point.

The elemental distribution map along the *Z*-axis showed interesting features: The Ti content was approximately 25 at.% up to 500 μm inside of the V layer from the Ti–6Al–4V/V interface, gradually reducing to 5 at.% from approximately 300 μm away from the interface. Severe penetration of Fe into the Cu and Monel layers can be observed, revealing an inhomogeneous distribution of the Cu concentration in the Cu and Monel layers. The presence of Fe beyond 50 at.% in the Cu and Monel alloy suggested complete mixing of the steel, Monel, and Cu layers during TIG welding. A similar observation could be made for Ni, which was distributed from the Cu layer to the steel upon mixing. Although these severe interlayer penetrations can be detrimental to the lamination, the layers and their thicknesses were carefully chosen to avoid the formation of IMCs. For example, Ti must be separated from Fe since it is prone to form Fe_2_Ti, which is detrimental to the fabrication process. Similarly, V must be separated from Fe since it accelerates sigma-phase formation.

**Figure 4:**
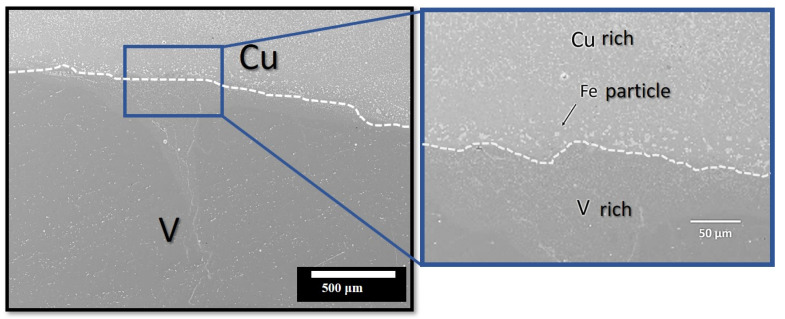
High-resolution SEM microstructure of V/Cu interlayer showing Fe precipitates.

In this study, the V concentration in the steel layer was negligible, and the opposite was true for Fe in the Ti–6Al–4V layer. Although these aspects of the elemental separation of Fe/Ti and V/Fe were the achievements of this study, the mixing of Fe in the Cu and Monel layers during TIG welding poses a serious threat to the fabrication process, as Cu cannot dissolve in Fe. Figure 4 shows that Fe precipitates out from the pure Cu layer, consistent with the EDS observations.

**Figure 5:**
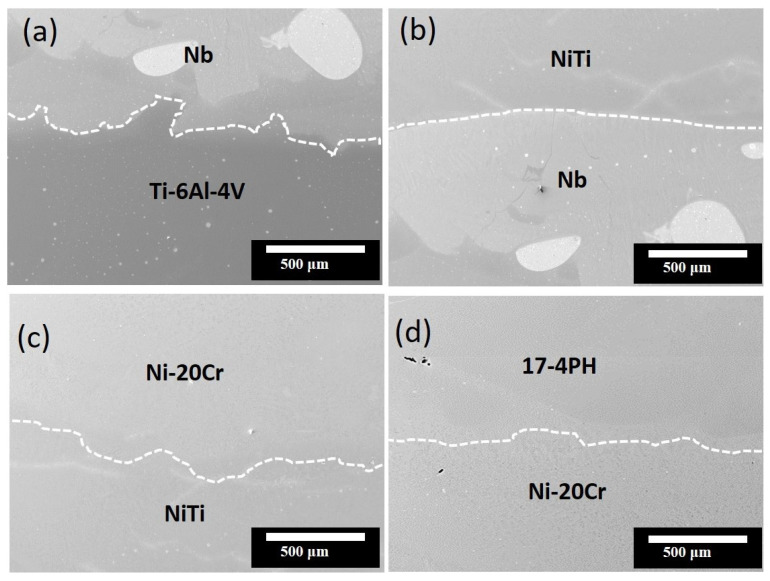
SEM microstructures of the morphology and cross-sectional image of Specimen 2 with the following layers: (**a**) Ti–6Al–4V/Nb, (**b**) Nb/Ni–Ti, (**c**) Ni–Ti/Ni–20Cr, and (**d**) Ni–20Cr/17-4PH.

**Figure 6:**
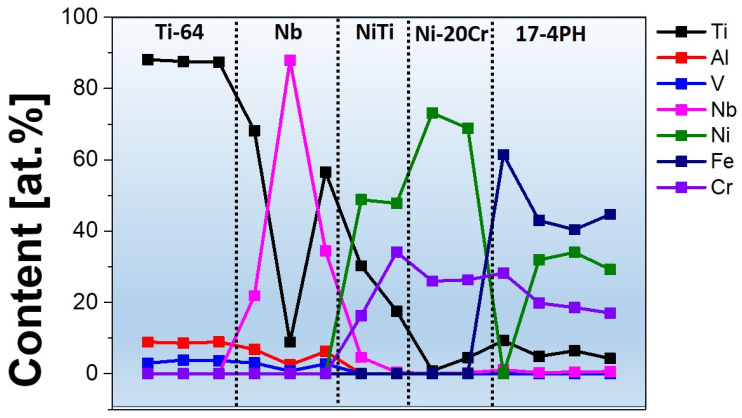
Elemental distribution throughout the interlayers of Specimen 2, obtained by EDS.

Figure 5 shows the morphology and cross-sectional image of Specimen 2, including the internal bonding of the Nb, Ni–Ti, and Ni–20Cr layers. The bonding between the dissimilar terminal alloys and interlayers was devoid of cracks, which is a significant result. Notably, each layer had the same variation of approximately 1000 μm. Figure 6 shows the EDS analysis of the elemental distributions along the fabrication direction from Ti–6Al–4V to 17-4PH steel.

The measurement process involved 16 evenly spaced points, and each point was measured for 10 min. The graph shows interesting patterns in the elemental distribution along the fabrication direction. It is apparent that there was significant penetration of Ti into the deposited Nb layer. The diffusion of Ti along the Nb and Ni–Ti layers followed a nonuniform pattern. Near the Nb interface, the Ti concentration was greater than 80 at.%. Subsequently, it reduced to 10 at.% in the middle of the Nb layers, and near the Ni–Ti interface, the Ti concentration increased to 55 at.%. The amount of Ti along the Ni–Ti layers was also found to vary, from 40 at.% to 20 at.%, along the Ni–Ti layers. However, there was no evidence of Ti in the Ni–Cr layers. Cr atoms diffused into the Ni–Ti layer, varying from 30 at.% near the interface to 15 at.% in the middle of Ni–Ti layer.

**Figure 7:**
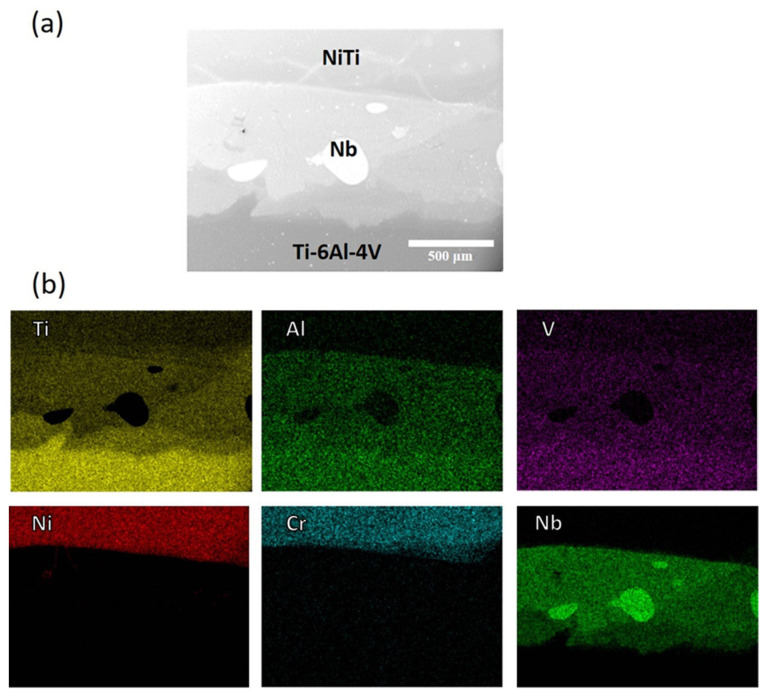
(**a**) SEM image of the Ti–6Al–4V/Nb/NiTi interlayers in Specimen 2, (**b**) Elemental distributions of Ti, Al, V, Ni, Cr and Nb obtained by EDS mapping.

The crystal structure is a significant factor influencing the diffusion of elements in a material. The crystal structures of adjacent layers can promote or hinder the diffusion process. The differences in the crystal structures of the Nb and Ni–Cr layers hindered the diffusion of Nb into the Ni–Cr layer. This highlights the importance of considering the crystal structure when designing layered materials for specific applications. Figure 7 shows the SEM image of the Ti–6Al–4V/Nb/Ni–Cr interlayers in Specimen 2, along with the elemental distributions obtained by EDS mapping, revealing regions in the Nb layer where the Nb content is very high, with negligible Ti content. This phenomenon is likely due to the phase separation in the Nb–Ti alloy. The Nb–Ti phase diagram indicates the presence of a miscibility gap in this alloy, resulting in the separation of the pure Nb and Nb–Ti phases. The presence of pure bcc Nb in some regions of the Nb layer was a significant finding. The presence of pure Nb within the Nb–Ti alloy can affect the mechanical properties of the material. This is due to the fact that the properties of pure Nb differ from those of the Nb–Ti alloy. For example, pure Nb is known for its excellent ductility and toughness, whereas Nb–Ti alloys are known for their high strength and stiffness. Therefore, it can be concluded from the X-ray mapping that the interlayer elements do not penetrate or mix throughout the fabrication route, thus avoiding the formation of detrimental IMCs. This was a significant result of this study.

### 3.2. Mechanical Properties

**Figure 8:**
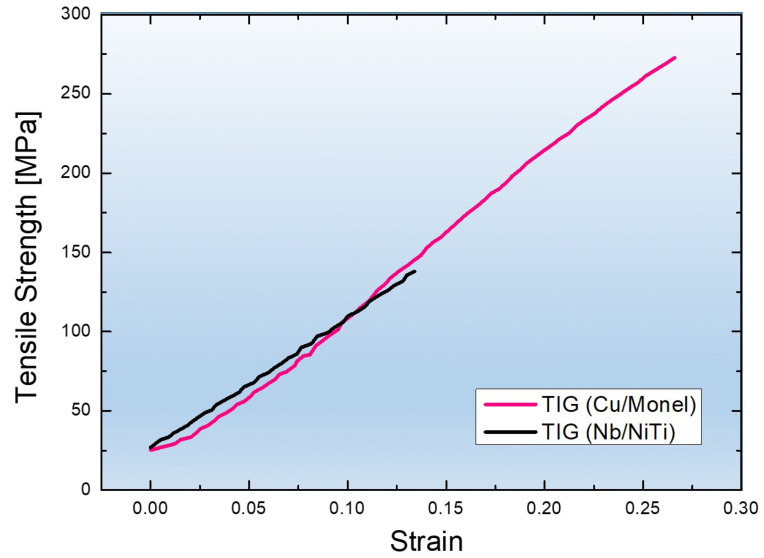
Engineering tensile stress–strain curves of Specimens 1 and 2 at 25 °C.

The tensile properties of Specimens 1 and 2 were investigated; Figure 8 shows the results. The tensile specimens were prepared with interlayers and a welded zone in the middle.

**Figure 9:**
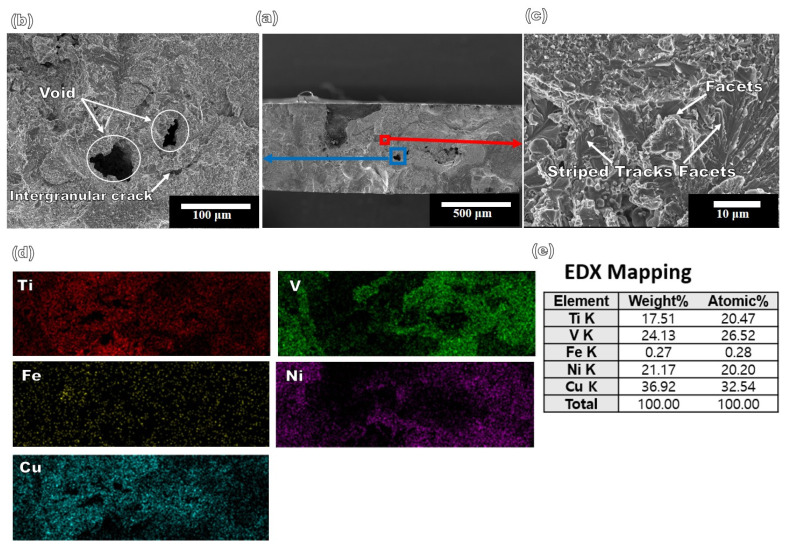
(**a**–**c**) SEM images of the fracture surface of Specimen 1, (**d**,**e**) EDS mapping and quantitative analysis of fracture surfaces.

Specimen 1 exhibited a tensile strength of 273 MPa, whereas Specimen 2 had a tensile strength of 137 MPa. The elongation values of Specimens 1 and 2 were 0.25 and 0.15, respectively. The obtained tensile strengths of Specimens 1 and 2 were significantly lower than the minimum strength required for wrought materials Ti–6Al–4V and 17-4PH, respectively. This could be due to the inhomogeneous distribution of the grain size and substantial defect formation associated with the nonequilibrium deposition and welding process. Furthermore, the interlayers in both the specimens contained pure elements, such as Cu and Nb, which have substantially lower tensile strengths than Ti–6Al–4V and 17-4PH. Repeated heating/cooling and stress-induced hardening of the interlayers and welded zones modified the microstructure of the specimens. The nonequilibrium deposition process used in this study can result in nonuniform microstructures and a significant number of defects, such as voids, cracks, and pores. These defects act as stress concentration sites and cause premature material failure. Notably, the tensile strength of Specimen 1 was two times that of Specimen 2, though the interlayer Nb had a relatively higher strength than Cu. This can be attributed to the diffusion of Fe in the Cu layers, which precipitated in the pure state and strengthened the Cu layer. Therefore, Cu acted as a better interlayer than Nb. However, it must be stated here that the nonequilibrium deposition and welding process used in this study should be further optimized to improve the tensile properties of the material. Figure 9 shows the SEM image of the tensile-tested fracture surface of Specimen 1, including the characteristics of the combined mixed mode of brittle and ductile fractures. The mixed mode can be attributed to the appearance of fibers and dimples in the microstructure, indicating ductile failure. The mechanism of this fracture involves three stages: Nucleation, growth, and propagation of voids.

**Figure 10:**
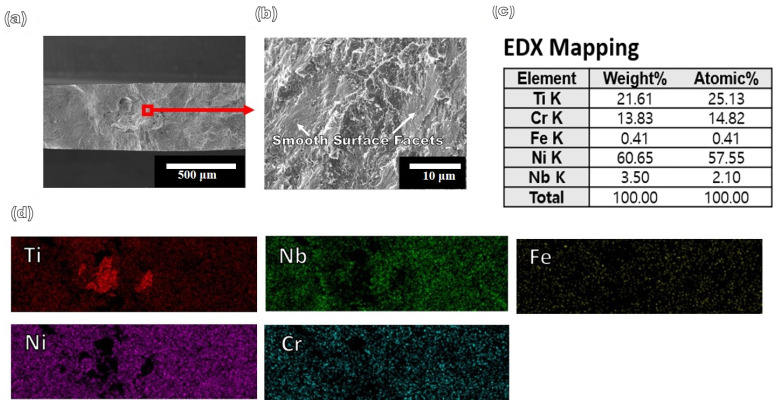
(**a**,**b**) SEM images of the fracture surface of Specimen 1, (**c**,**d**) EDS mapping and quantitative analysis of fracture surfaces.

When the materials were loaded, voids were formed in regions where there existed heterogeneous sites of inclusions, pores, and blow holes. The presence of micropores on the fracture surface further indicated a lower ductility. Micropores are small voids that can form due to various reasons, including inadequate processing, thermal stress, and material defects. The presence of cracks and facets was considered as evidence of a brittle failure. Cracks were nucleated by the presence of heterogeneities, such as depressions, striations, holes, and steps, which served as stress concentration sites. These cracks propagated along their specified crystallographic directions under the release of strain energy, which was the driving force for the propagation. Inter-crystalline cracks formed since the voids were preferentially located along the grain boundaries. Brittle failure was also predicted when the interatomic bonds were broken at maximum loading, resulting in reinforcement pull-out, crack nucleation and propagation, and final rupture. Figure 9d shows the EDS mapping of the fracture surface of Specimen 1 which revealed that the fracture occurred at the Cu layers, as the fracture surface contained 37 at.% Cu. The Ti, V, and Ni contents were 17, 24, and 21 at.%, respectively. Moreover, the Ti-enriched region of the fracture surface was devoid of V and Ni. The V and Ni separated from Ti under the application of stress during tensile testing. However, Cu was distributed throughout the matrix. The phase separation of Ti from the rest of the matrix can lead to a change in the microstructure, making it potentially vulnerable to stress applications. V enrichment of 12 at.% could be observed in the Cr fracture surface of the as-built tensile-tested specimen, whereas it was 6 at.% in the heat-treated specimen before testing. Cr atoms were segregated around the cleavage planes of the fracture surface, resembling a phase separation during tensile testing. Figure 10 shows the fracture surface of the tensile-tested Specimen 2. The fracture surface predominantly showed cleavage facets and a few dimples, indicative of a brittle fracture. Brittle fracture occurs when a material fails without significant plastic deformation.

Cleavage facets are smooth surfaces resulting from the separation of crystal planes along specific weak planes. In this case, it is likely that the micropores are related to processing or material defects that contribute to the low ductility of the material. The observed facets can be classified into two types based on their surface features: Smooth surface facets and facets with striped tracks. Smooth surface facets were observed all over the surface and were considered to be the source of crack initiation. In contrast, the facets with striped tracks were randomly distributed on the surface. The striped tracks are likely due to slip bands formed during crack propagation. Slip bands are regions of highly localized plastic deformation induced ahead of the crack tip during crack propagation. These were due to the stresses generated by the crack tip, which resulted in a dislocation movement and plastic deformation. The slip bands can be straight or curved and can have various orientations. In this case, the striped tracks were likely the result of the straight slip bands formed during crack propagation. Interestingly, the smooth surface facets had a distinct grain-shaped boundary, whereas the striped surface facets lacked this feature. This is likely due to the grain boundary blocking effect. During the first facet formation, the slip band was smaller than the grain size since the first facet was formed inside the grain. Consequently, the grain boundary blocked the slip band, resulting in a clear boundary between the facet and the grain. The grain-boundary blocking effect was most prominent when the length of the slip band was in or below the order of the grain size. Therefore, the smooth surface facet is believed to be the first facet generated, with the grain-boundary blocking effect playing a significant role. As the cracks continued to propagate, the slip band during striped-facet formation elongated, thereby reducing the blocking effect of the grain boundary. Therefore, the predominant observation of cleavage facets and the presence of micropores suggest that the material exhibited a brittle fracture behavior. Figure 10c,d show the EDS analysis of the fracture surface of Specimen 2, revealing that fracture occurred at the Ni–Ti layers, as the fracture surface contained 57 at.% Ni. The Ti and Cr contents of the fracture surfaces were 25 and 13 at.%, respectively. However, the Ti atoms were segregated and clustered on the fracture surface. Interestingly, the Ti-enriched region of the fracture surface was completely devoid of Ni, clearly indicating the phase separation of Ti from the Ni matrix. This could lead to microstructural changes and make the surface potentially vulnerable to stress application. Moreover, the clear surface facet of the fractured surface was completely devoid of Ti, whereas the striped surface facet was enriched with Ti. Therefore, it can be concluded that the segregation of Ti in the secondary facets is responsible for the lower ductility of Specimen 2.

## 4. Conclusions

- TIG welding can be effectively used to fabricate complex specimens made of Ti–6Al–4V/V/Cu/Monel400/17-4PH and Ti–6Al–4V/Nb/Ni–Ti/Ni–Cr/17-4PH.
- The specimens were effectively bonded internally with no cracks, exhibiting a substantial tensile strength.
- Specimen 1 exhibited a higher tensile strength (273 MPa) than Specimen 2 (137 MPa). Their elongation values were 0.25 and 0.15, respectively.
- The interface microstructures of the interlayers were analyzed using FE-SEM. The specimens were found to be effectively bonded internally, with no cracks in the bonding between the interlayers and dissimilar terminal alloys.
- Severe interlayer penetration of Fe and Ni was found in the Cu and Monel alloys, and the diffusion of Ti along the Nb and Ni–Ti layers followed a nonuniform distribution, which is detrimental to the lamination.
- Our findings suggest that the elemental composition and distribution of a material at the point of fracture play a significant role in determining its failure mechanism.

## Data Availability

Data of this thesis are available on-request.

## References

[1] Campo K.N., Campanelli L.C., Bergmann L., dos Santos J.F., Bolfarini C. (2014). Microstructure and Interface Characterization of Dissimilar Friction Stir Welded Lap Joints between Ti–6Al–4V and AISI 304. Mater. Des. (1980–2015).

[2] Chu Q., Tong X., Xu S., Zhang M., Yan F., Cheng P., Yan C. (2020). The Formation of Intermetallics in Ti/Steel Dissimilar Joints Welded by Cu-Nb Composite Filler. J. Alloys Compd..

[3] Jin P., Liu Y., Sun Q., Li J., Chen K., Hou S., Yang H., Zhou Y., Feng J. (2020). Wetting Mechanism and Microstructure Evolution of TC4/304 Stainless Steel Joined by CMT with an Assisted Hybrid Magnetic Field. J. Alloys Compd..

[4] Adomako N.K., Lewandowski J.J., Arkhurst B.M., Choi H., Chang H.J., Kim J.H. (2022). Microstructures and Mechanical Properties of Multi-Layered Materials Composed of Ti-6Al-4V, Vanadium, and 17–4PH Stainless Steel Produced by Directed Energy Deposition. Addit. Manuf..

[5] Choo W., Ebrahimian M., Choi K., Kim J.H. (2023). Influence of Heat Treatment on the Microstructure and Hardness of 17-4PH Stainless Steel Fabricated Through Direct Energy Deposition. Met. Mater. Int..

[6] Park C.W., Adomako N.K., Lee M.G., Kim J.H., Kim J.H. (2021). Interfacial Structure and Pore Formation Mechanism during Laser Cladding of Pure Vanadium on Ti-6Al-4V Alloy. Int. J. Refract. Met. Hard Mater..

[7] Woong Park C., Narayan Hajra R., Kwabena Adomako N., Choo W., Yang S.-M., Seo S.-J., Kim J.H. (2023). Additive Manufacturing of Ti-6Al-4V/V-Interlayer/17-PH Steel Functionally Graded Material Using Angular and Spheroidal V Powders. Mater. Lett..

[8] Akbari Mousavi S.A.A., Sartangi P.F. (2008). Effect of Post-Weld Heat Treatment on the Interface Microstructure of Explosively Welded Titanium–Stainless Steel Composite. Mater. Sci. Eng. A.

[9] Wang T., Zhang B., Feng J., Tang Q. (2012). Effect of a Copper Filler Metal on the Microstructure and Mechanical Properties of Electron Beam Welded Titanium–Stainless Steel Joint. Mater. Charact..

[10] Chen S., Zhang M., Huang J., Cui C., Zhang H., Zhao X. (2014). Microstructures and Mechanical Property of Laser Butt Welding of Titanium Alloy to Stainless Steel. Mater. Des..

[11] Xia Y., Dong H., Zhang R., Wang Y., Hao X., Li P., Dong C. (2020). Interfacial Microstructure and Shear Strength of Ti6Al4V Alloy/316 L Stainless Steel Joint Brazed with Ti33.3Zr16.7Cu50−xNix Amorphous Filler Metals. Mater. Des..

[12] Yang L., Jiang X., Sun H., Song T., Mo D., Li X., Luo Z. (2020). Interfacial Reaction and Microstructure Investigation of TC4/V/Cu/Co/316L Diffusion-Bonded Joints. Mater. Lett..

[13] Li B., Chen Z., He W., Zhou T., Wang Y., Peng L., Li J., Liu Q. (2019). Effect of Titanium Grain Orientation on the Growth of Compounds at Diffusion Bonded Titanium/Steel Interfaces. Mater. Charact..

[14] Kahraman N., Gülenç B., Findik F. (2005). Joining of Titanium/Stainless Steel by Explosive Welding and Effect on Interface. J. Mater. Process. Technol..

[15] Dong H., Yu L., Deng D., Zhou W., Dong C. (2015). Effect of Post-Weld Heat Treatment on Properties of Friction Welded Joint Between TC4 Titanium Alloy and 40Cr Steel Rods. J. Mater. Sci. Technol..

[16] Xia Y., Dong H., Li P. (2020). Brazing TC4 Titanium Alloy/316L Stainless Steel Joint with Ti50-XZrxCu39Ni11 Amorphous Filler Metals. J. Alloys Compd..

[17] Zhang Y., Zhou J., Sun D., Li H. (2020). Two Pass Laser Welding of TC4 Titanium Alloy to 301L Stainless Steel via Pure V Interlayer. J. Mater. Res. Technol..

[18] Zhang Y., Sun D.Q., Gu X.Y., Duan Z.Z., Li H.M. (2018). Nd:YAG Pulsed Laser Welding of TC4 Ti Alloy to 301L Stainless Steel Using Ta/V/Fe Composite Interlayer. Mater. Lett..

[19] Zhang Y., Zhou J., Sun D., Li H. (2020). Three-Pass Laser Welding of Ti Alloy-Stainless Steel Using Nb and Ni Interlayers. J. Mater. Res. Technol..

[20] Pugacheva N.B., Makarov A.V., Senaeva E.I., Volkova E.G. (2019). Crystallization of Dissimilar Ti/Cu/Steel Laser Welds. J. Cryst. Growth.

[21] Hao X., Dong H., Yu F., Li P., Yang Z. (2021). Arc Welding of Titanium Alloy to Stainless Steel with Cu Foil as Interlayer and Ni-Based Alloy as Filler Metal. J. Mater. Res. Technol..

[22] Hao X., Li P., Xia Y., Dong H., Wang P., Yan D. (2019). Microstructure and Mechanical Properties of Dissimilar TC4 Titanium Alloy/304 Stainless Steel Joint Using Copper Filler Wire. Met. Mater. Trans. A.

[23] Li J., Liu Y., Gao Y., Jin P., Sun Q., Feng J. (2020). Benefits of Interfacial Regulation with Interlayers in Laser Welding Ti6Al4V/316L Steel. Opt. Laser Technol..

[24] Chu Q., Zhang M., Li J., Yan C. (2017). Experimental and Numerical Investigation of Microstructure and Mechanical Behavior of Titanium/Steel Interfaces Prepared by Explosive Welding. Mater. Sci. Eng. A.

